# Fracture healing in a mouse model of Hajdu–Cheney-Syndrome with high turnover osteopenia results in decreased biomechanical stability

**DOI:** 10.1038/s41598-023-38638-0

**Published:** 2023-07-14

**Authors:** Tobias Malte Ballhause, Shan Jiang, Weixin Xie, Jan Sevecke, Christine Dowling, Tobias Dust, Sabine Brandt, Peter R. Mertens, Timur Alexander Yorgan, Thorsten Schinke, Karl-Heinz Frosch, Anke Baranowsky, Johannes Keller

**Affiliations:** 1 Department of Trauma and Orthopedic Surgery, University Medical Center Hamburg-Eppendorf, 20246 Hamburg, Germany; 2 Department of Nephrology and Hypertension, Diabetes and Endocrinology, Otto-von-Guericke University, 39120 Magdeburg, Germany; 3 Department of Osteology and Biomechanics, University Medical Center Hamburg-Eppendorf, 20246 Hamburg, Germany; 4 Department of Trauma Surgery, Orthopedics and Sports Traumatology, BG Hospital Hamburg, 21033 Hamburg, Germany

**Keywords:** Trauma, Musculoskeletal system

## Abstract

Notch signaling regulates cell fate in multiple tissues including the skeleton. Hajdu–Cheney-Syndrome (HCS), caused by gain-of-function mutations in the *Notch2* gene, is a rare inherited disease featuring early-onset osteoporosis and increased risk for fractures and non-union. As the impact of Notch2 overactivation on fracture healing is unknown, we studied bone regeneration in mice harboring a human HCS mutation. HCS mice, displaying high turnover osteopenia in the non-fractured skeleton, exhibited only minor morphologic alterations in the progression of bone regeneration, evidenced by static radiological and histological outcome measurements. Histomorphometry showed increased osteoclast parameters in the callus of HCS mice, which was accompanied by an increased expression of osteoclast and osteoblast markers. These observations were accompanied by inferior biomechanical stability of healed femora in HCS mice. Together, our data demonstrate that structural indices of bone regeneration are normal in HCS mice, which, however, exhibit signs of increased callus turnover and display impaired biomechanical stability of healed fractures.

## Introduction

Impaired fracture healing poses a large clinical problem as treatment options remain limited^1^. Despite state-of-the art surgical and non-surgical care, non-union of fractured bone occurs in 10–15% of affected patients. Non-unions are associated with high socioeconomic costs and a significant reduction in quality of life, as affected patients often are not able to work, exhibit prolonged hospitalization time, suffer from pain, and require multiple revision surgeries^2^. Several risk factors have been identified that predispose to disturbed fracture healing. Apart from lifestyle factors, metabolic diseases and certain drugs, specific genetic mutations have been described that result in an increased fracture risk and poor healing outcomes^3^.

In this regard, the Hajdu–Cheney-Syndrome (HCS) represents a rare autosomal-dominant disorder that is characterized by acro-osteolysis and early-onset osteoporosis^4^. HCS is caused by nonsense or deletion mutations within exon 34 of the gene encoding the Notch2 receptor, resulting in excessive activation of Notch2 signaling^5^. Affected individuals have a high fracture risk, with fractures often occurring in childhood. Although clear evidence is missing due to a lack of sufficient clinical data, fracture non-union also seems to occur more frequently in HCS according to several case reports^6–11^. The phenomenon of fracture non-union is most commonly reported in childhood but is also found in individuals of other age groups^12^. Most importantly, while acro-osteolysis and early-onset osteoporosis can be treated by anti-resorptive therapy, fracture non-union may be severely incapacitating in affected patients, and is thus considered indeed more problematic than most other HCS sequelae^13^.

Mechanistically, Notch2 belongs to the Notch receptor family, comprised of Notch1-4, which are expressed in almost all organ systems and play a pivotal role in cell fate decision by coordinating cell proliferation, differentiation, and apoptosis^14^. The Notch signaling pathway determines organ development via cell fate regulation^15^. It is mainly a cell-to-cell signaling pathway, as both ligand and receptor are expressed on cell surfaces^16,17^. Notch receptors represent transmembrane proteins and are highly conserved during evolution with structural and functional similarities in all mammals^18^. Each of the four receptors can interact with all hitherto identified canonical ligands (Jagged1, Jagged2, Delta-like ligand (Dll)-1, -3, and -4). It is known that the function of the different Notch receptors is not redundant, underlined by the fact that their expression is unequally distributed throughout different tissues and cell types^19^.

In bone, numerous functions have been assigned to Notch receptors and their ligands, including osteoblast proliferation and differentiation, matrix mineralization, as well as osteoclast recruitment and cell fusion^20^. Physical force on the receptor results in exposure of the intracellular binding sites and thus activation of the canonical arm of the signaling pathway^21^. With this functional mechanism, the Notch signaling pathway plays a crucial role in bone remodeling and the skeleton’s adaptation to changing mechanical stress^22^. In this regard, Notch2 is expressed both in osteoclasts and osteoblasts^23^. While total inactivation of Notch2 causes embryonic lethality in mice, selective inactivation of Notch2 in osteoclasts is not associated with skeletal abnormalities. In contrast, mice with an osteoblast-specific deletion of Notch2 demonstrate an increase in trabecular bone formation in the proximal femur and distal tibia^24^.

To understand the impact of Notch2 signaling on bone turnover in HCS in more depth, we previously generated a HCS mouse model by introducing a pathogenic mutation (6272delT) in the Notch2 gene^25^. Like HCS patients, the model displayed osteopenia in both the axial skeleton and the long bones at 3, 6, and 12 months of age, but not acro-osteolysis at any timepoint studied. While ex vivo osteoblast and osteoclast differentiation were not affected in bone marrow cells, mutant mice show elevated bone formation and resorption indices in vivo. As a potential explanation, an unbiased RNA sequencing approach identified *Tnfsf11* (encoding pro-resorptive RANKL) and *Il6* (encoding pro-inflammatory interleukin 6) as potential mediators, indicating a net pro-resorptive expression pattern triggering high bone turnover.

As a high risk for fractures and, subsequently, for non-unions were described as the most severe complications of HCS, the current study was designed to test the hypothesis that fracture healing is impaired in HCS mice^26^. Therefore, we employed a standardized fracture model (femur osteotomy stabilized by an external fixator) and studied bone regeneration at early, intermediate, and late stages using radiological, histological, molecular and biomechanical outcome measures^27^. Our results show that in the employed HCS mouse model of HCS, normal morphological healing signs are observed throughout all stages of bone regeneration. However, the regenerating bone of HCS mice also displays indices of increased callus turnover, ultimately resulting in inferior biomechanical stability of the healed bones.

## Results

### Notch2 is expressed in callus tissue

**Figure 1:**
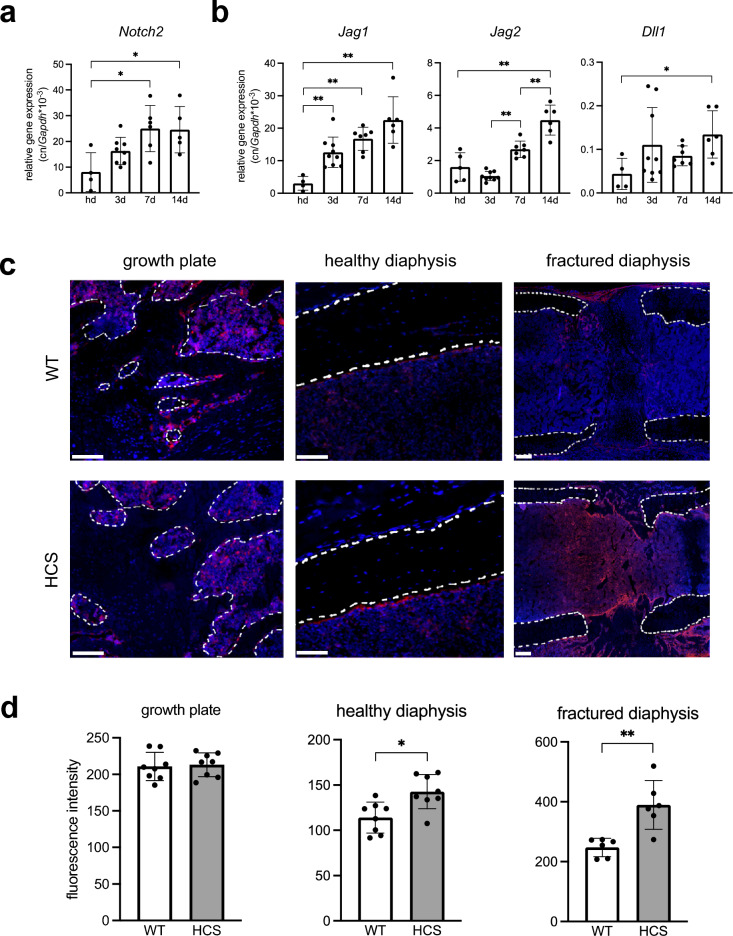
Notch2 is expressed in callus tissue. (**a**) Expression of Notch2 in the fracture callus of WT mice at the indicated time points. Hd = non-fractured, healthy diaphysis (**b**) Expression of the Notch receptor ligands Jagged1 (*Jag1*), Jagged2 (*Jag2*), and Delta-like-canonical-Notch-ligand-1 (*Dll1*) in the same samples. (**c**) Representative images of immunofluorescent stainings of Notch2 in the indicated femoral compartments. Upper row: non-fractured and fractured femur of WT mice. Lower row: non-fractured and fractured femur of HCS mice. Scale bar = 100 μm. Dotted white lines show trabecular bone (growth plate), non-fractured cortical bone (diaphysis), or fracture ends (diaphysis), respectively. (**d**) Quantification of respective immunofluorescent signal intensities of Notch2. For (**a**, **b**, **d**), non-parametric Mann–Whitney U test, *p < 0.05; **p < 0.005. Data are shown as mean with standard deviation. Data points indicate individual measurements from each mouse.

To characterize the role of Notch2 signaling in bone regeneration, we first monitored expression of the *Notch2* gene in the fracture callus of WT mice which were subjected to a femoral osteotomy. Here, *Notch2* was steadily expressed at all stages of bone regeneration, with similar expression levels as compared to independent, non-fractured femora (Fig. 1a). Monitoring the expression of canonical Notch2 ligands, we found the expression of *Jagged1, Jagged2,* as well as *Delta-like-canonical-Notch-ligand-1* to be significantly induced during the course of fracture healing (Fig. 1b). Next, we monitored Notch2 expression on protein level in the femur of WT and HCS mice using immunofluorescent visualization of a Notch2-specific antibody. The growth plate and healthy cortical bone were analyzed in the non-fractured, contralateral femur of mice with a femoral osteotomy (Fig. 1c). In WT mice, Notch2 expression was found in cells lining trabecular bone as well as in the bone marrow adjacent to both the growth plate´s proliferation zone (dotted line) and the cortical bone (dotted line) of the non-fractured femoral shaft. In the callus of WT mice, strong signals were detected in the periosteum around the fracture site as well as in ossification centers 14 days after osteotomy. We next examined whether increased levels of Notch2 are present in the non-fractured and fractured femora of HCS mice. Immunofluorescence demonstrated strongly enhanced signals in cortical bone of HCS mice. Moreover, in HCS mice the Notch2 signal within the callus area was also greatly amplified 14 days after osteotomy, indicative of enhanced Notch2 activation. Subsequent quantification of Notch2 signal intensity confirmed a significant increase both in the non-fractured and fractured femoral diaphysis of mutant mice (Fig. 1d).

### Regular radiological healing process in HCS mice with femoral fracture

**Figure 2:**
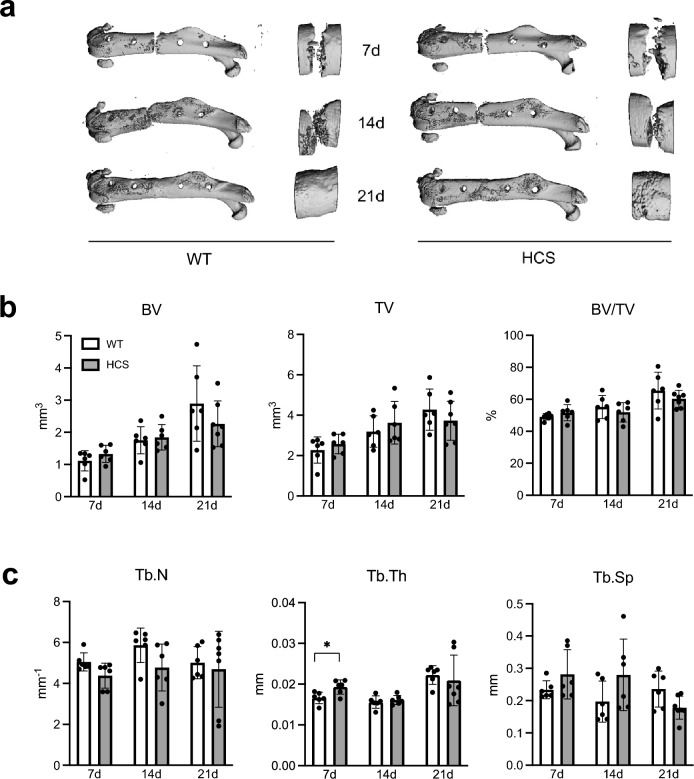
Regular radiological healing process in HCS mice with femoral fracture. (**a**) Representative µCT images of the callus of WT and HCS mice 7, 14, and 21 days after osteotomy. Images were generated by SCANCO Medical microCT Analysis Software V6/10–2009, https://www.scanco.ch/images/Brochures/microct_sw_.v8.pdf, *Brüttisellen, Switzerland* (**b**) Quantification of bone volume (BV), tissue volume (TV) and bone volume per tissue volume (BV/TV). (**c**) Quantification of trabecular numbers (Tb.N.), trabecular thickness (Tb.Th.), and trabecular separation (Tb.Sp.) in the same groups. Non-parametric Mann–Whitney U test, *p < 0.05. Data are shown as mean with standard deviation. Data points indicate individual measurements from each mouse.

As these findings suggested that bone regeneration may be affected by excessive activation of Notch2, we next monitored fracture healing in WT and HCS mice 7, 14, and 21 days after a femoral osteotomy. These time points correspond to the inflammatory, the soft callus, and the hard callus stages of bone healing. µCT-scanning of fractured femora did not reveal any morphological differences in the healing course of WT and HCS mice (Fig. 2a). Quantification of radiologic callus parameters showed that bone and tissue volume of the callus progressively increased during healing in mice of both genotypes, indicating morphologically mostly normal bone healing in HCS mice (Fig. 2b). Assessment of callus architecture revealed a significantly elevated trabecular thickness in HCS mice 7 days following surgery, while trabecular numbers and trabecular separation were unaltered throughout the entire course of bone healing (Fig. 2c).

### Normal histological callus maturation but increased osteoclast numbers in HCS mice

**Figure 3:**
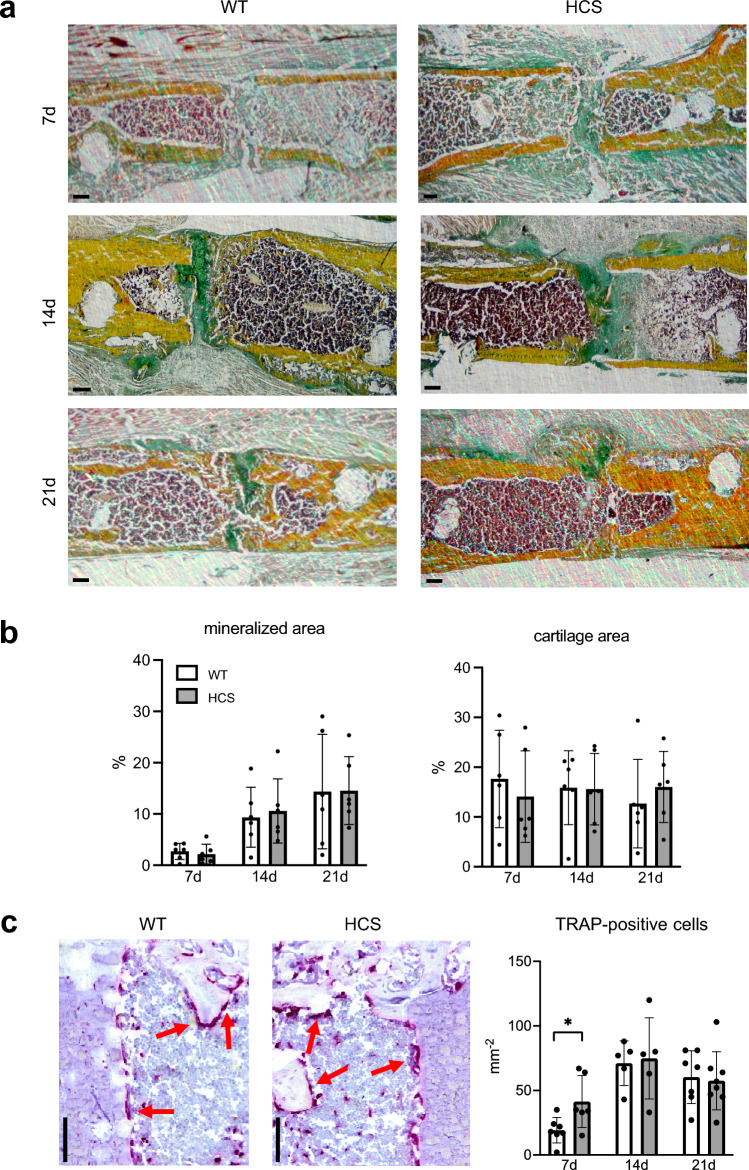
Normal histological callus maturation but increased osteoclast numbers in HCS mice. (**a**) Representative callus sections (Movat’s Pentachrome staining) of WT and HCS mice at the indicated time points (yellow = mineralized bone; green = cartilage; reddish/brown = bone marrow). Scale bar = 100 μm. Images were captured with CellSens Standard V4.1, Olympus, https://www.olympus-lifescience.com/de/software/cellsens/*Tokio, Japan.* (**b**) Histomorphometric quantification of static callus parameters (cartilage and mineralized area) in the same mice. (**c**) Representative images of tartrate-resistant acid phosphatase stainings 21 days after femoral osteotomy, osteoclasts are stained in red. The corresponding quantitative analysis is displayed on the right. Scale bar = 100 μm. For (**a**–**c**), non-parametric Mann–Whitney U test, *p < 0.05. Data are shown as mean with standard deviation. Data points indicate individual measurements from each mouse.

To characterize the tissue composition of the healing callus in both groups, we next performed histomorphometry of undecalcified callus sections stained with Movat´s pentachrome (Fig. 3a). In this staining, mineralized bone appears yellow whereas cartilage is green. For analysis of mineralized callus components, only newly formed bone was considered. Seven days after osteotomy, 2.7% (± 1.6) of the region of interest was newly formed bone in WT and 2.2% (± 1.9) in HCS mice (Fig. 3b). Subsequently, further bone growth bridging the osteotomy gap was observed, resulting in 14.3% (± 11.2) in WT and 14.6% (± 6.6) in HCS mice 21 days following surgery. Assessing cartilage distribution, representing an essential intermediate tissue component that is later replaced by mineralized bone, we observed an average of 17.6% (± 9.8) in WT and 14.1% (± 9.2) in HCS mice at day 7 after osteotomy. The cartilage parameters showed only minor alterations at 14 and 21 days after surgery and did not differ significantly between WT and mutant mice. Finally, as increased osteoclastogenesis is observed in the skeleton of HCS mice, we determined osteoclast numbers in the callus of both groups using tartrate-resistant acid phosphatase (TRAP) activity stainings. Significant increased osteoclast numbers were counted in HCS per tissue area in the callus of HCS mice 7 days after osteotomy (WT: 19 ± 9.9 osteoclasts; HCS: 41 ± 20, *p* = 0.03). This phenomenon was not seen at day 14 or day 21 in the histomorphometric quantification, at which osteoclast numbers were unaltered (Fig. 3c).

### Adequate fracture union in HCS mice

**Figure 4:**
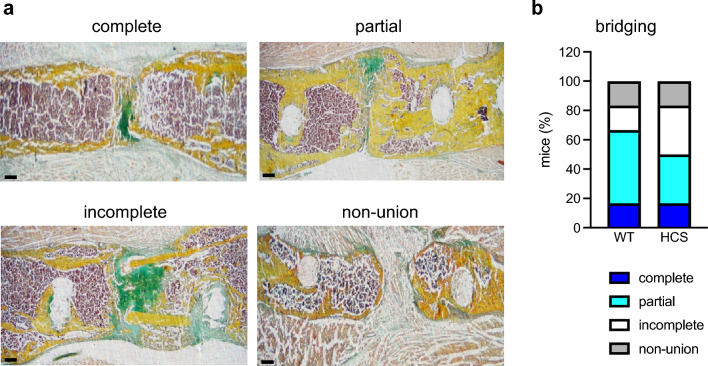
Adequate fracture union in HCS mice. (**a**) Representative callus sections (Movat’s Pentachrome staining) of four exemplary types of osseous bridging to score fracture union 21 days after osteotomy: complete bridging (all four cortices bridged by callus), partial bridging (two to three cortices bridged by callus), incomplete bridging (callus present, but no bridging visible), and non-union (rounded cortices, minimal presence of callus). Images were captured with CellSens Standard V4.1, Olympus, https://www.olympus-lifescience.com/de/software/cellsens/*Tokio, Japan.* (**b**) Semi-quantitative scoring of osseous bridging in WT and HCS mice 21 days after osteotomy. n = 6 mice per group.

Apart from the formation of mineralized and cartilaginous tissue in the callus, osseous bridging of the fracture gap is important for adequate bone repair. Therefore, we assessed fracture union in undecalcified callus sections stained with Movat´s Pentachrome 21 days after osteotomy (Fig. 4a). In both groups with n = 6 mice per genotype, 1 mouse showed complete union. Partial bridging was observed in 3 WT mice and in 2 HCS mice (Fig. 4b). Incomplete fracture bridging was seen in 1 WT mouse and 2 HCS mice. Total non-union occurred in 1 mouse of each group. Together, this semi-quantitative scoring indicated that osseous bridging of the fracture gap does not differ overall between WT and HCS mice.

### HCS mice display indices of increased callus remodeling on gene expression level

**Figure 5:**
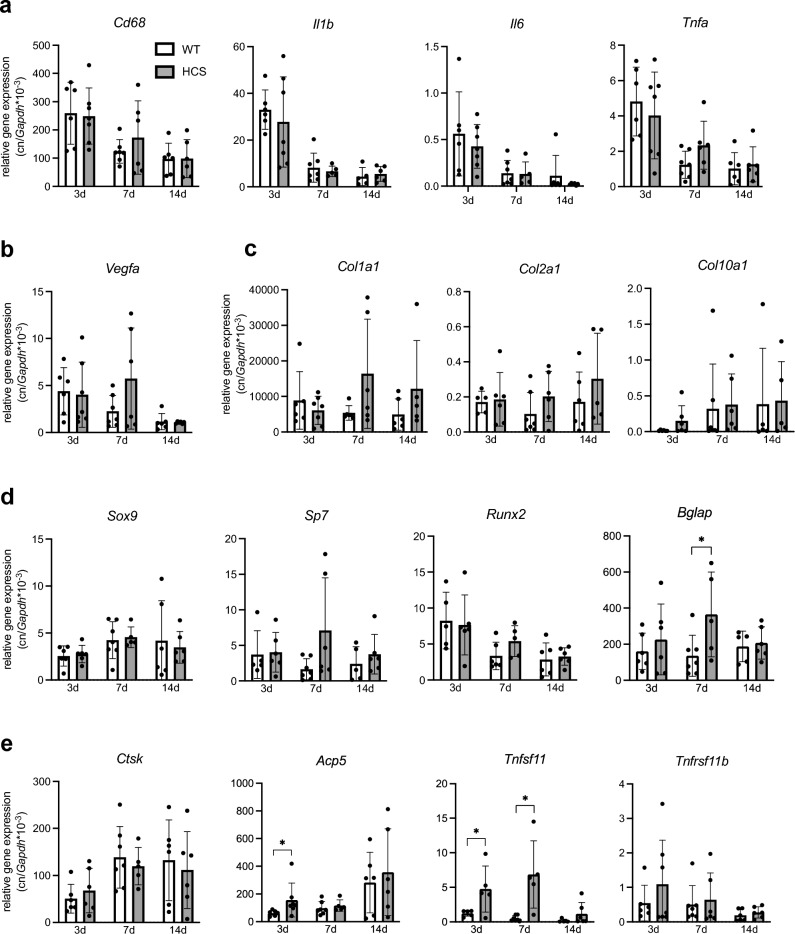
HCS mice display indices of increased callus remodeling on gene expression level. (**a**) qRT-PCR expression analysis (virtual copy numbers per *Gapdh)* of the indicated inflammation-related genes in the callus of WT and HCS mice 3, 7, and 14 days after osteotomy (*Cd68* = cluster of differentiation 68; *Il1b* = interleukin-1 beta; *Il6* = interleukin-6; *Tnfa* = tumor necrosis factor alpha). (**b**) Expression of an angiogenesis-associated gene (*Vegfa* = vascular endothelial growth factor a) and (**c**) collagens (*Col1a1* = Collagen Type I alpha 1 chain; *Col2a1* = collagen type II alpha 1 chain; *Col10a1* = collagen type X alpha 1 chain) are depicted. (**d**) Chondroblast markers (*Sox9* = SRY-Box transcription factor 9) and expression of osteoblast marker genes (*Sp7* = *Sp 7 transcription factor*, also known as *osterix*; *Runx2* = runt-related transcription factor 2*; Bglap* = bone gamma carboxyglutamate protein, alternatively termed osteocalcin) were analyzed. (**e**) Expression of genes associated with osteoclastogenesis (*Ctsk* = cathepsin k; *Acp5* = acid phosphatase 5, tartrate resistant; *Tnfsf11* = tumor necrosis factor ligand superfamily, member 11, encoding RANKL; *Tnfrsf11b* = tumor necrosis factor receptor superfamily, member 11b, encoding osteoprotegerin). For (**a**–**e**), non-parametric Mann–Whitney U test, *p < 0.05. Data are shown as mean with standard deviation. Data points indicate individual measurements from each mouse. Group sizes may vary due to sample loss during RNA processing.

To characterize the fracture healing process on the molecular level, we again subjected WT and HCS mice to a femoral osteotomy and extracted callus RNA 3, 7, and 14 days after surgery. Monitoring the expression of cytokines regulating the post-traumatic inflammatory response critical for adequate healing, no significant differences were observed in the case of the macrophage marker cluster of differentiation 68 (*Cd68*), interleukin-1 beta (*Il1b*), interleukin-6 (*Il6*) or tumor necrosis factor alpha (*Tnfa*) (Fig. 5a). Likewise, no alteration in the expression of vascular endothelial growth factor A (*Vegfa*), regulating angiogenesis in callus tissue, as well as the cartilage markers SRY-box transcription factor 9 (*Sox9*) and the collagens type 1 alpha 1 (*Col1a1*), type 2 alpha 1 (*Col2a1*) and type 10 alpha1 *(Col10a1)* were detected (Fig. 5b,c). Regarding markers of bone formation, we observed a tendency towards increased expression in the case of osterix (*Sp7*) and runt-related transcription factor 2 (*Runx2*) in HCS mice 7 days following surgery (Fig. 5d). Moreover, *Bglap*, encoding the key osteoblast marker osteocalcin, was significantly overexpressed at the same time point in HCS mice. In terms of bone resorption, cathepsin K (*Ctsk*) expression was unaltered, however we observed a significant induction of the osteoclast marker acid phosphatase 5, tartrate resistant (*Acp5*) in HCS mice 3 days after osteotomy (Fig. 5e). As a potential explanation, a strikingly enhanced expression of receptor Activator of NF-κB Ligand (*Rankl; Tnfsf11*), essential for osteoclast formation, was detected 3 and 7 days after surgery, while its decoy receptor osteoprotegerin (*Opg*; *Tnfrsf11b*) was unaltered.

### Impaired biomechanical stability of healed femora in HCS mice

**Figure 6:**
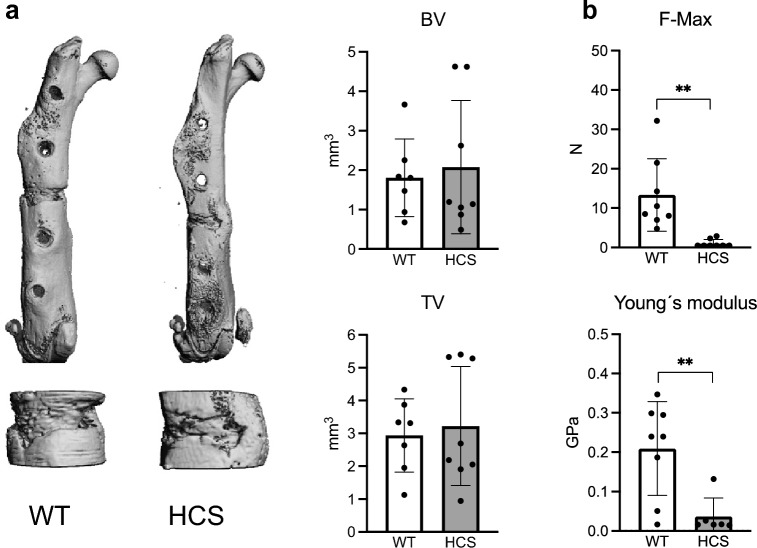
Impaired biomechanical stability of healed femora in HCS mice. (**a**) Representative µCT images of the healing femur of WT and HCS mice 28 days after osteotomy. Upper row: overview. Lower row: detailed image of callus. Quantification of bone volume (BV) and tissue volume (TV) in the same mice. Images were generated by SCANCO Medical microCT Analysis Software V6/10–2009, https://www.scanco.ch/images/Brochures/microct_sw_v8.pdf, *Brüttisellen, Switzerland* (**b**) Quantification of mechanical properties of the healing femora as determined by three-point-bending test in the same animals. F-Max = maximum force that the bone could withstand; Young’s modulus = stiffness along the axis. Non-parametric Mann–Whitney U test, **p < 0.005. Data are shown as mean with standard deviation. Data points indicate individual measurements from each mouse.

These results indicated that cellular callus remodelling is elevated in HCS mice. Therefore, we finally asked whether this would affect the late-stage healing outcome and overall biomechanical stability. To this end, we subjected WT and HCS mice to a femoral osteotomy and performed µCT-scanning and biomechanical testing 28 days after surgery. µCT-scanning demonstrated no significant alteration in bone volume and tissue volume of the fracture callus between WT and HCS mice (Fig. 6a). However, biomechanical testing using three-point bending test revealed a significantly reduced force to structural fracture in HCS mice (Fig. 6b). This was accompanied by a reduced Young’s modulus, indicative of impaired structural elasticity of the healed callus.

## Discussion

HCS is caused by nonsense or deletion mutations within the Notch2 gene, resulting in excessive activation of Notch2 signaling and subsequent acro-osteolysis and early-onset osteoporosis^23^. Based on a series of clinical case reports, it is now considered that HCS is not only associated with disturbances in bone remodeling, but potentially also with an increased risk for fracture non-union. In this experimental study, we found that, despite only slight morphological abnormalities, the healing bone of HCS mice is characterized by indices of increased callus turnover and inferior biomechanical stability in a highly standardized and clinically relevant fracture model.

Increasing evidence demonstrates that Notch signaling plays divergent yet essential roles in the regulation of bone turnover^22^. HCS, caused by activating mutations of Notch2, is a human genetic disease with distinct skeletal manifestations, underlining the importance of Notch signaling in the adult skeleton. HCS was first described as a cranioskeletal dysplasia by Hajdu and Kauntze^28^. It is now clear that HCS is an autosomal dominant disorder, which leads to early-onset osteoporosis and pathognomonic acro-osteolysis. Reports also include short stature, vertebral abnormalities, long bone deformities and joint hypermobility as clinical manifestations of the syndrome^29^. Although it is not entirely clear to date, experimental data suggest that an increased osteoclast formation, accompanied by an elevated bone formation, provide the mechanistic bases for the skeletal complications of HCS^30,31^. In this regard, our previously generated HCS mouse model showed high turnover osteopenia with elevated bone resorption and formation. Osteoclasts and osteoblasts derived from HCS mice did not show a cell-autonomous defect, however we found an increased expression of the pro-osteoclastic factors Rankl and Il6 in HCS bone marrow cells. As the high turnover and skeletal pathologies could be fully normalized by treatment with the bisphosphonate alendronate, we concluded that overactivation of Notch2 favors a pro-resorptive expression pattern triggering high bone turnover.

Similar to bone remodeling, bone regeneration following a fracture is a complex process, involving a broad array of different cell types and signaling cascades^32^. In this regard, Notch signaling is also considered to be of central importance during bone healing^33^. During endochondral ossification, a process critical for adequate bone regeneration, the expression of all four Notch receptors is induced. In line with this, we detected increased expression of *Notch2* and the canonical Notch ligands *Jag1*, *Jag2*, and *Dll1* during the course of fracture healing compared to non-fractured bone. Moreover, Notch signaling in skeletal progenitors was shown to be critical for bone healing. In an elegant study by Wang et al., deletion of the Notch effector RBPjk in bone marrow stromal cells resulted in fracture non-union, whereas Notch inactivation in osteoblasts or chondrocytes had no effect^34^. Likewise, Wang et al. observed enhanced Notch1 signaling in osteochondroprogenitor cells during bone regeneration. It was associated with reduced cartilage formation, elevated mineralized callus tissue volume, and stronger bones 3 weeks after fracture. Thereafter, the authors indirectly inhibited Notch signaling by systemic application of the γ-secretase inhibitor DAPT, and found that callus formation was increased through the promotion of MSC differentiation, resulting in an overall accelerated fracture healing^34^. Thus, despite an increasing number of reports on the role of Notch receptors during bone regeneration, the complex cellular interactions and the multitude of involved cell types require further investigation to fully understand the role of Notch receptors in fracture healing.

Based on other and our own previous findings, in the current study we hypothesized that fracture healing is impaired in HCS mice. To this end, we first characterized Notch2 expression on protein level in both non-fractured and fractured femora of HCS mice and WT littermates. While Notch2 expression was found in cells lining trabecular bone and the cortical bone of the non-fractured femoral shaft, strong signals in the periosteum around the fracture site as well as in ossification centers within the callus were observed in WT mice with osteotomies. Notch2 signals were significantly intensified in HCS mice, especially in the regenerating callus, indicating successful overexpression of Notch2 protein in this model and pointing towards a potential impact on fracture healing. However, using radiological and static histological measurements, we observed only minor structural changes in bone regeneration in HCS mice, with an overall normal morphologic healing outcome. In this regard, µCT-scanning demonstrated that callus mineralization and fracture union did not differ between WT and mutant animals, except for a slight increase in trabecular thickness in HCS mice 7 days after osteotomy. Histologically, the distribution of mineralized and cartilaginous tissues developed in a similar fashion over time in both WT and HCS mice. Interestingly, the only detectable abnormality was an increase in osteoclast numbers in the healing callus of HCS mice at day 7 following osteotomy, whereas at later timepoints, no significant differences were detected. On the molecular level, this finding was accompanied by an increased expression of the osteoclast marker *Acp5*, potentially explained by overexpression of the key osteoclastogenic cytokine *Rankl* in the callus of HCS mice at early and intermediate healing stages. Together with the tendency towards increased expression of osteoblast markers in the callus of HCS mice, reaching statistical significance in the case of osteocalcin, these findings indicate that callus remodeling is overall increased in mutant animals. Despite normal morphologic fracture healing in HCS mice, this high callus turnover was associated with inferior biomechanical stability at late healing stages, as evidenced in three-point-bending tests. As a potential explanation, disorganization of collagen fibers or irregular deposition of calcium phosphate crystals in the callus of HCS mice may be considered and require further study.

To the best of our knowledge, the impact of an overall high bone turnover on fracture healing is still unclear. For example, Kondo et al. observed accelerated fracture healing in mice with increased circulating c-type natriuretic peptide and generalized high bone turnover^35^. In contrast, in an experimental model of hyperparathyroidism, characterized by excessive bone turnover, fracture healing was delayed^36^. It is thus possible that ultra-structural abnormalities, resulting from high callus remodeling and not evident in standard radiological and histological assays, underlie the inferior biomechanical stability of healed femora in HCS mice.

## Limitations of this study

Our study has several limitations. First, this study used only male mice at the age of 12–14 weeks, and it remains uncertain, if the findings can also be generalized to older mice and to females. Second, as the employed HCS mouse model is characterized by global overactivation of Notch2 signaling, our approach does not allow to delineate the exact cellular and molecular mechanisms underlying the observed phenotypical changes. Third, in this highly standardized model of stiff external fixation of a defined osteotomy, callus formation is not as pronounced as in other models with less rigid fixation. Therefore, different results may be obtained in closed fracture models with intramedullary fixation, where higher callus volumes can be observed. Finally, our results imply that radiologic indices of bone regeneration may appear normal in HCS patients with healing fractures despite potentially impaired biomechanical stability. However, since results from animal fracture models cannot be directly translated into clinical practice, caution needs to be taken when clinically assessing the healing progress in affected HCS patients.

In conclusion, we report here that in a murine mode of HCS, bone regeneration shows normal morphological signs of healing progression. In contrast, the callus of HCS mice shows cellular and molecular indices of increased turnover, which is associated with impaired biomechanical stability at late healing stages. Further clinical studies are warranted to characterize the course and outcome of bone regeneration in HCS patients with fractures.

## Methods

### Animals

For all experiments in this study, male HCS mice (*Notch2*^+*/HCS*^; B6; 129-Notch2tm1Hhtg/Uke) and WT littermates were used. The generation of *Notch2*^+*/HCS*^ mice was described previously^25^. HCS mice harbor a 6272delT mutation in the intracellular domain of the Notch2 gene, impeding ubiquitination and subsequent degradation of the Notch-intracellular domain-2 (NICD2). All mice were kept at a 12 h light /12 h dark cycle in a SPF facility and fed a standard diet and water ad libitum. All animal experiments were approved by the local legal representative animal rights protection authorities (N121/2020) and performed adherent to the policies and principles established by the animal Welfare Act (Federal Law Gazett I, p.1094) and the national institutes of health guide for care and use of laboratory animals. In total, 88 mice were operated and analyzed for the experiments.

### Surgical procedure

Male mice at the age of 12–14 weeks with a body weight > 20 g received a femoral osteotomy, stabilized by an external fixator (RISystem, *Landquart, Switzerland*), as previously described^27^. Briefly, following anesthesia with isoflurane, a lateral approach on the right hind leg´s thigh was made to expose the femur and the external fixator was placed on the anterior-lateral femur. A mid-diaphyseal osteotomy was made using a 0.66-mm Gigli wire saw (RISystem), resulting in an osseous defect of 0.7 mm in the femur shaft. The external fixator was placed strictly parallel to the femur and provided rigid fixation with angular stability of the construct. The wound was closed with Mersilene 4–0 suture (Johnson & Johnson, *New Brunswick, NJ, USA*). Prior to surgery Clindamycin (150 mg kg^−1^) and buprenorphine (0.1 mg kg^−1^) were administered. For optimal pain relief, the mice received metamizole (4 g/l) via the drinking water for 7 days post-operatively. Mice were sacrificed at 3, 7, 14, 21, and 28 days after surgery as indicated by cardial exsanguination under general anesthesia. Fractured femora were harvested for subsequential measurements. For each experiment, 6–9 mice of each genotype were employed per time point as indicated by the individual data points in respective graphs. Sample size was calculated a priori to reduce as many animals as possible used for experiments, but still generating statistical power. Differences in group sizes are explained by loss or destruction of individual samples during processing.

### Micro-computed tomography

The fractured femur was fixed in 4% paraformaldehyde (PFA) over night and washed with phosphate-buffered saline (PBS). The femur was scanned by µCT (vivaCT 80, SCANCO Medical, *Brüttisellen, Switzerland*) with A voxel size of 15.6 µm at 70 kVp, 113 µA and 400 ms integration time. Three-dimensional (3D) images were generated via µCT Ray V4.0-4 (Scanco Medical). Morphological parameters were evaluated using μCT Evaluation Program V6.6 (Scanco Medical). All analyses were performed on a volume of interests (VOI) compromising 100 slices including the callus and cortices. Data is reported according to the guidelines for tissue imaging by the American Society of Bone and Mineral Research^37^.

### Histological analysis

After µCT scanning, the samples were processed according to a modified Kawamoto frozen section protocol^32,38^. Briefly, fractured femora were infiltrated in an ascending sugar gradient (10%, 20% and 30% each for 24 h at 4 °C), embedded in SCEM medium (Section Lab Co Ltd., *Hiroshima, Japan*) and stored at − 80 °C until further processing. The cryo-blocks were cut longitudinally in transversal plane in 7 µm sections using a cryotome (Cryostat M630, Medite*, Burgdorf, Germany*). The sections of the same area (transversal visible bone marrow with 4 cortices) were mounted on microscope slides using cryofilm (Cryofilm type II C, Section Lab Co Ltd.). Movat’s Pentachrome and tartrate-resistant acid phosphatase (TRAP) staining was carried out. For histomorphometry, images were captured using BX-50 (Olympus, *Tokio, Japan*) microscope and evaluated with OsteoMeasure program (Osteometrics, *Atlanta, GA, USA*). A region of interest (ROI) measuring 1 × 1 mm was defined on the osteotomy site. On Movat´s Pentachrome slides, the yellow stained, newly formed bone was encircled. Remainings of the cortical bone, which existed prior to the fracture, were excluded from the analysis. In parallel, the green stained cartilage area was encircled. The selected areas were measured and divided by the area of the ROI, resulting in the relative percentages of newly mineralized and cartilaginous tissues, respectively. Similarly, TRAP staining was evaluated with OsteoMeasure by manually counting TRAP-positive cells in the same RIO covering the entire osteotomy site.

Callus bridging was evaluated with the following scoring: complete bridging = all four cortices bridged by callus; partial bridging = two to three cortices bridged by callus; incomplete bridging = callus present, but no bridging visible; non-union = rounded cortices, minimal presence of callus. Scoring was performed independently by two blinded reviewers.

### Immunofluorescence

To localize Notch2 expression on protein level in non-fractured and fractured bone, immunofluorescence staining of Notch2 was carried out. Cyro-sections were blocked in 3%BSA/5% Donkey Serum/PBS and incubated with anti-Notch2 (1:100, R&D, AF5196) overnight. Following subsequent washing, secondary antibody (1:500, Life technologies, A21098) was applied and Fluromount-G with DAPI (Thermo Fisher Scientific, *Waltham, MA, USA*) was used for mounting. Images were acquired using a spinning disk confocal microscope (Aurox Ltd, *Oxfordshire, UK*). In ImageJ (U. S. National Institutes of Health, *Bethesda, Maryland, USA*), a ROI measuring 1 × 1 mm was defined. It fully covered the osteotomy site and respectively the growth plate in non-fractured controls. Red and blue channels were then selected to specify the immunoflurocent antibody and the counterstaining. The intensity of red within the ROI was counted.

### Gene expression analysis

For gene expression analysis, an independent set of WT and HCS animals was used. Mice were euthanatized after 3, 7 and 14 days for extraction of callus tissue. For the analysis of non-fractured femoral diaphysis (data exclusively displayed in Fig. 1a,b), the femoral midshafts of another independent group of WT mice (C57Bl/6J genetic background, 14 weeks old) were harvested. In this group, sample size also varies due to only limited available sample material.

All samples were processed using a standardized RNA extraction protocol. Briefly, the tissue was homogenized in TRIzol (Invitrogen, *Waltham, MA, USA*) with an UltraTurrax (Sigma-Aldrich Chemie GmbH, *Taufkirchen, Germany*). Following phase separation and RNA precipitation using chloroform and isopropanol, RNA was further purified with the Nucleospin RNA II kit (Macherey–Nagel, *Düren, Germany*). RNA quality and integrity were assessed using Nanodrop 2000 (Nanodrop Technology Inc., Thermo Scientific). Complementary DNA was synthesized using First Strand cDNA Synthesis Kit (New England Biolabs, *Ipswich, MA, USA*). Quantitative real-time PCR (qRT-PCR) was carried out using TaqMan Assay-on-Demand primer sets (*Jag1, Jag2, CD68, IL-1b, IL-6, Tnfa, Vegf, Sox9, Col1a1, Col2a1, Col10a1, Sp7, Runx2, Bglap, Ctsk, Acp5, RankL, Opg*) supplied by Applied Biosystems (*Waltham, MA, USA*) or SYBR Green (*Dll1)*. For SYBR Green assays, the following primer sequences were used: *Gapdh* forward 5′-TGCACCACCAACTGCTTAG-3′; *Gapdh* reverse 5′-GGATGCAGGGATGATGTTC-3′; *Dll1* forward 5′-CTATCCGGACCCCAATTCCC-3′; *Dll1* reverse 5′-CACGGAGAGGTGAGTGTCTC-3′. Gene expression was normalized to the housekeeper gene Glyceraldehyde-3-phosphate dehydrogenase (*Gapdh*) and calculated by the ΔΔCT method.

### Biomechanics

Three-point-bending test was conducted on healed femora harvested at day 28 after osteotomy using a universal testing machine Z2.5/TN1S and the testXpert software (both Zwick Roell, *Ulm, Germany*). In brief, the femur was horizontally mounted and centrally positioned with the patella groove facing upwards onto the two bars of the device at a support distance of 7 mm. A constant bending load of 0.05 m/s was applied onto the callus site and the load–displacement data was recorded until failure. The derived parameters were calculated in the testXpert software.

### Statistical analysis

All data reported in the manuscript are presented as individual data points with mean ± standard deviations. The statistical analysis was performed using Graph-PadPrism Software 9 (*La Jolla, CA, USA*). Parametric distribution was tested with the Shapiro–Wilk test and data was assessed by Mann–Whitney U-test. *p* < 0.05 was considered statistically significant.

### Ethical approval

All animal experiments were approved by the local legal representative animal rights protection authorities *(Behörde für Justiz und Verbraucherschutz der Freien und Hansestadt Hamburg)* and trials were listed as N121/2020. All experiments were performed in accordance with relevant guidelines and regulations, respecting the European Directive 2010/63/EU and performed adherent to the policies and principles established by the animal Welfare Act (Federal Law Gazett I, p. 1094). All methods are reported according to the ARRIVE guidelines.

## Data Availability

The datasets generated during and analyzed during the current study are available from the corresponding author on reasonable request.

## Abbreviations

Acp5: Acid phosphatase 5
Alpl: Alkaline phosphatase
Bglap: Bone gamma carboxyglutamate protein
BMD: Bone mineral density
BS: Bone surface area
BV: Bone volume
Col: Collagen
Ctsk: Cathepsin K
Dll: Delta-like ligand
HCS: Hajdu–Cheney-Syndrome
IL: Interleukin
µCT: Micro-computed tomography
NICD: Notch intracellular domain
qRT-PCR: Quantitative real-time polymerase chain reaction
Runx2: Runt-related transcription factor 2
Sp7: Transcription factor 7; Osterix
Sox9: Sex-determining region Y box transcription factor 9
TMD: Tissue mineral density
TNFα: Tumor necrosis factor-α
Tb. N.: Trabecular number
Tb. Sp.: Trabecular space
Tb. Th.: Trabecular thickness
TV: Tissue volume
VEGFA: Vascular epithelial growth factor A
WT: Wild type
